# The Precision Revolution in Hematologic Malignancies: A Decade of Transformative Immunotherapies and Targeted Agents

**DOI:** 10.3390/jcm14248896

**Published:** 2025-12-16

**Authors:** Ghaith K. Mansour, Ahmad W. Hajjar, Muhammad Raihan Sajid

**Affiliations:** 1 Department of Pharmaceutical Sciences, College of Pharmacy, Alfaisal University, Riyadh 11533, Saudi Arabia; gkmansour@alfaisal.edu; 2 College of Medicine, Alfaisal University, Riyadh 11533, Saudi Arabia; awhajjar@alfaisal.edu; 3 Department of Pathology, College of Medicine, Alfaisal University, Riyadh 11533, Saudi Arabia

**Keywords:** hematologic malignancies, targeted therapy, immunotherapy, precision medicine, combination strategies

## Abstract

This review describes the dramatic transformation that has occurred in the last ten years in the therapeutic landscape for hematologic malignancies, such as leukemias, lymphomas, myelomas, and myelodysplastic syndromes. Treatment paradigms have quickly changed from depending solely on cytotoxic chemotherapy to embracing precision medicine, driven by a previously unprecedented understanding of disease biology and precise molecular changes. The development of powerful immunotherapies (such as CAR T-cell therapy and bispecific antibodies) and innovative targeted agents (like BTK inhibitors, BCL-2 inhibitors, and immunomodulatory medications) is at the heart of this revolution. In addition to evaluating new and synergistic combination strategies, this paper examines the clinical utility, efficacy, and recent developments of these novel agents. It also addresses important issues like managing acquired drug resistance, minimizing financial burden, and adapting clinical trial designs to keep pace with innovation. These advancements are collectively redefining clinical practice, leading to deeper and more durable responses, and significantly improving the prognosis and quality of life for patients.

## 1. Introduction

Hematologic malignancies encompass a diverse spectrum of cancers affecting blood-forming tissues, including leukemias, lymphomas, myelomas, and myelodysplastic syndromes, collectively accounting for approximately 10% of all new cancer diagnoses globally [1].

The past decade has witnessed an unprecedented transformation in the therapeutic landscape of these diseases, driven by advances in our understanding of disease biology, the development of targeted therapies, and the emergence of novel immunotherapeutic approaches [2]. Traditional treatment paradigms, which relied heavily on cytotoxic chemotherapy and radiation therapy, have increasingly given way to precision medicine approaches that target specific molecular alterations and harness the immune system’s capacity to recognize and eliminate malignant cells [3]. The regulatory landscape has adapted accordingly, with the FDA approving 66 new drugs for hematologic malignancies between 2019 and 2024, with 53% of these approvals based on early-phase clinical trial data [4].

This therapeutic renaissance has been characterized by several paradigm-shifting developments. CAR-T cell therapy has evolved from an experimental treatment to a standard-of-care option, with six CAR-T products now FDA-approved for various hematologic indications [5]. BTK inhibitors (BTK = Bruton’s tyrosine kinase) have revolutionized the treatment of B-cell malignancies, particularly chronic lymphocytic leukemia (CLL) and mantle cell lymphoma, with newer-generation agents offering improved safety profiles [6]. The BCL-2 inhibitor genetical has demonstrated remarkable synergy with hypomethylating agents in acute myeloid leukemia (AML), leading to its adoption as first-line therapy for older patients [7].

This review provides a comprehensive analysis of the most significant therapeutic advances in hematologic malignancies, examining the clinical evidence supporting these innovations, their integration into current treatment algorithms, and the challenges that remain. We will explore recent developments in immunotherapy, targeted therapy, and combination strategies while discussing emerging resistance mechanisms and future therapeutic directions. Unlike recent reviews focusing on single modalities or specific diseases, this work provides a cross-disease synthesis of immunotherapy and targeted therapy advances, integrates regulatory and economic perspectives, and highlights future directions in trial design and biomarker development.

## 2. Recent Developments in Hematologic Malignancy Therapeutics

### 2.1. CAR-T Cell Therapy: Expanding Applications and Enhanced Efficacy

Chimeric antigen receptor T-cell therapy represents one of the most transformative advances in cancer treatment, with particular impact in hematologic malignancies [8]. The field has progressed rapidly from initial proof-of-concept studies to widespread clinical implementation, with ongoing research focused on expanding target antigens, improving manufacturing processes, and reducing toxicities.

Recent clinical trials have demonstrated the efficacy of CAR-T therapy in earlier treatment lines, most notably with the FDA approval of axicabtagene ciloleucel (axi-cel) and lisocabtagene maraleucel (liso-cel) for second-line treatment of large B-cell lymphoma [9]. The landmark TRANSFORM study showed that liso-cel significantly improved event-free survival compared to standard salvage chemotherapy followed by autologous stem cell transplant, with a median event-free survival not reached versus 2.4 months (HR 0.349, 95% CI 0.254–0.478, *p* < 0.0001) [9].

Manufacturing innovations have focused on improving CAR-T cell expansion, persistence, and functionality [10]. CRISPR-based gene editing approaches are being explored to enhance CAR-T cell performance by deleting immune checkpoint receptors [11]. The development of “off-the-shelf” allogeneic CAR-T products aims to address manufacturing delays and expand patient access, though challenges related to graft-versus-host disease and immune rejection remain to be fully resolved.

Toxicity management has evolved significantly, with improved understanding of cytokine release syndrome (CRS) and immune effector cell-associated neurotoxicity syndrome (ICANS) [8]. Real-world data demonstrate that CRS occurs in approximately 40–50% of patients receiving currently approved CAR-T products, with grade 3–4 CRS in less than 10% of cases [12]. Prophylactic strategies, including tocilizumab administration and corticosteroids, have shown promise in reducing severe toxicity without compromising efficacy [13].

### 2.2. BTK Inhibitors: Second-Generation Agents and Beyond

The BTK inhibitor landscape has evolved considerably since the initial approval of ibrutinib, with second-generation agents offering improved selectivity and reduced off-target effects [14]. Acalabrutinib and zanubrutinib have demonstrated superior safety profiles compared to ibrutinib in head-to-head clinical trials, with significantly reduced rates of atrial fibrillation, hypertension, and bleeding complications.

The ELEVATE-RR trial demonstrated that acalabrutinib was associated with significantly fewer cardiovascular adverse events compared to ibrutinib in patients with CLL, with atrial fibrillation occurring in 9.4% versus 16.0% of patients, respectively (*p* = 0.02) [6]. Similarly, the ALPINE study showed that zanubrutinib had superior progression-free survival and fewer side effects compared to ibrutinib [15].

Resistance to BTK inhibitors has emerged as a significant clinical challenge, primarily mediated by mutations at the C481 binding site of BTK or downstream signaling components such as PLCγ2 [16]. These observations have driven the development of non-covalent BTK inhibitors, including pirtobrutinib, which maintains activity against C481S-mutant BTK [17]. Clinical trials of pirtobrutinib have shown overall response rates of approximately 70% in patients with prior BTK inhibitor exposure, regardless of resistance mechanism [18].

Novel approaches to BTK targeting include BTK degraders, which promote the degradation of the entire BTK protein rather than simply inhibiting its kinase activity. These agents demonstrate activity against both wild-type and mutant forms of BTK and represent a promising strategy for overcoming resistance [19].

### 2.3. Venetoclax and Combination Strategies in AML

The BCL-2 inhibitor venetoclax has fundamentally altered the treatment landscape for AML, particularly in older adults who are ineligible for intensive chemotherapy [7].

Triplet combinations incorporating venetoclax have shown particular promise in molecularly defined subgroups of AML [20]. The addition of gilteritinib to venetoclax and azacitidine in FLT3-mutated AML achieved a complete remission rate of 90% in newly diagnosed patients, with 65% of evaluable patients achieving FLT3-ITD measurable residual disease negativity. These results support the concept of mutation-directed therapy selection and the potential for cure in historically high-risk subgroups [20].

IDH inhibitor combinations with venetoclax have also demonstrated encouraging results [21]. Network meta-analysis suggests that venetoclax plus azacitidine may provide superior overall survival compared to IDH inhibitor combinations in IDH-mutated AML, though direct comparative studies are needed to confirm these findings [22]. The landmark VIALE-A trial established venetoclax plus azacitidine as a new standard for unfit AML, demonstrating superior overall survival versus azacitidine alone (14.7 vs. 9.6 months; HR 0.66). This regimen has since been integrated into global guidelines and inspired triplet combinations in molecularly defined subgroups.

### 2.4. Bispecific Antibodies in Multiple Myeloma

Bispecific antibodies have emerged as a transformative therapy for multiple myeloma, with three agents now FDA-approved for heavily pretreated patients [23]. Teclistamab, elranatamab, and talquetamab target BCMA or GPRC5D, redirecting T-cells to myeloma cells and achieving remarkable response rates in triple-class-exposed patients.

Clinical trials have demonstrated overall response rates of 60–74% with these agents, with complete response rates ranging from 25 to 50% [24]. The MajesTEC-1 study of teclistamab showed an overall response rate of 63% in heavily pretreated patients, with a median progression-free survival of 11.3 months. Real-world evidence has largely confirmed these efficacy results [25]. while providing insights into optimal patient selection and toxicity management [26]. Resistance mechanisms to bispecific antibodies are becoming better understood, with antigenic changes being a primary mechanism of escape [27]. T-cell characteristics, including the presence of regulatory T-cells and expression of exhaustion markers, significantly impact response to bispecific antibody therapy [28]. Strategies to overcome resistance include combination with immunomodulatory agents, immune checkpoint inhibitors [29].

### 2.5. Menin Inhibitors: Targeting Transcriptional Dependencies

Menin inhibitors represent a novel class of targeted therapies for acute leukemias driven by KMT2A rearrangements or NPM1 mutations [30]. These agents work by disrupting the interaction between menin and the KMT2A protein complex, leading to downregulation of HOXA9 and MEIS1 transcription factors that are essential for leukemic cell survival.

Revumenib became the first FDA-approved menin inhibitor based on results from the AUGMENT-101 study, which demonstrated an overall response rate of 68% in heavily pretreated KMT2A-rearranged acute leukemia patients [31]. The median duration of response was 9.1 months, with differentiation syndrome being the most significant toxicity concern, occurring in approximately 16% of patients [31].

Ziftomenib, another menin inhibitor in clinical development, has shown similar efficacy [32]. Combination strategies with menin inhibitors are being actively investigated, including combinations with DOT1L inhibitors, which target a complementary chromatin-modifying enzyme in the same pathway [33].

### 2.6. Therapeutic Advances by Disease Category

#### 2.6.1. Acute Myeloid Leukemia

AML has experienced a therapeutic renaissance with the approval of multiple targeted agents over the past five years [34]. Beyond venetoclax combinations, FLT3 inhibitors have established a clear benefit in FLT3-mutated disease [35].

Midostaurin in combination with standard chemotherapy demonstrated improved overall survival in newly diagnosed FLT3-mutated AML [36], while gilteritinib showed superiority over salvage chemotherapy in relapsed/refractory disease [37].

IDH inhibitors have provided targeted therapy options for the approximately 20% of AML patients harboring IDH1 or IDH2 mutations [38]. Ivosidenib and enasidenib have demonstrated efficacy in relapsed/refractory disease, with ongoing studies evaluating their integration into frontline therapy. The combination of IDH inhibitors with intensive chemotherapy has shown promising preliminary results, with high rates of morphologic remission and IDH mutation clearance [39].

Emerging targets in AML include MCL-1, which represents a potential mechanism of venetoclax resistance. MCL-1 inhibitors are in early-phase clinical development and may provide synergistic activity when combined with venetoclax [40,41]. Collectively, these advances have transformed AML into a molecularly stratified disease, where targeted agents now offer meaningful survival benefits across genetic subsets.

The principles of precision oncology have been validated in pediatric malignancies through landmark biomarker-driven trials. Initiatives such as the INFORM (Individualized Therapy For Relapsed Malignancies in Childhood) registry and MAPPYACTS have demonstrated the feasibility of comprehensive molecular profiling (including whole-genome/exome and RNA sequencing) to identify actionable alterations and guide targeted therapy in children with relapsed/refractory cancers, including AML [42,43]. These studies have successfully matched a significant proportion of pediatric patients to targeted agents (e.g., FLT3 or IDH inhibitors, MEK inhibitors) based on molecular findings, leading to clinically meaningful responses. This paradigm reinforces the universal applicability of biomarker-driven treatment strategies and highlights the critical need to expand access to molecular testing and novel therapies across all age groups.

#### 2.6.2. Chronic Lymphocytic Leukemia

CLL treatment has been revolutionized by the availability of targeted therapies that provide superior efficacy compared to traditional chemoimmunotherapy while offering improved tolerability profiles [6]. The combination of BTK inhibitors with venetoclax has emerged as a highly active regimen capable of achieving deep remissions, including undetectable measurable residual disease in a significant proportion of patients [44].

The CLL14 study demonstrated that venetoclax plus obinutuzumab achieved superior progression-free survival compared to chlorambucil plus obinutuzumab in previously untreated CLL patients, with the additional benefit of fixed-duration therapy [45]. The CAPTIVATE study showed that ibrutinib plus venetoclax achieved undetectable measurable residual disease in 77% (peripheral blood) and 60% (bone marrow) of the patients after 12 cycles of combination therapy.

For patients with high-risk features such as del(17p) or TP53 mutations, both BTK inhibitors and venetoclax-based regimens have demonstrated superior efficacy compared to chemotherapy. These patients, who previously had extremely poor outcomes with conventional therapy, can now achieve durable remissions with targeted approaches [46]. Thus, CLL management has evolved toward chemotherapy-free, fixed-duration regimens capable of achieving deep molecular remissions, even in high-risk disease.

The success of precision medicine in CLL is part of a broader trend in hematologic oncology, further exemplified by pediatric precision medicine programs. Studies like iTHER (Innovative Therapies for Children with Cancer) and others have shown that comprehensive molecular characterization can reveal targetable pathways in relapsed pediatric leukemias and lymphomas, leading to the compassionate use of targeted agents (e.g., BTK inhibitors in certain contexts) [47]. These efforts underscore the translational potential of molecular diagnostics and the importance of extending biomarker-driven trial designs to encompass both adult and pediatric populations to accelerate therapeutic discovery.

#### 2.6.3. B-Cell Lymphomas

**Figure 1:**
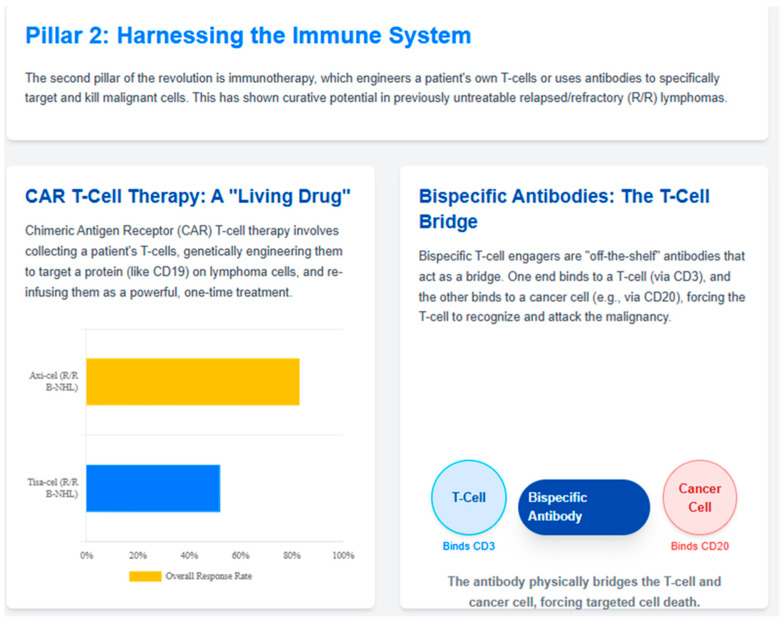
Figure 1 outlines the mechanisms of CAR T-cell therapy and bispecific antibodies, emphasizing their role in redirecting immune responses against malignant cells.

The historical introduction of rituximab, a chimeric anti-CD20 monoclonal antibody, in the late 1990s marked the first successful application of therapeutic monoclonal antibodies in oncology and revolutionized the treatment of B-cell non-Hodgkin lymphomas (NHL) [48]. Its addition to CHOP chemotherapy (R-CHOP) significantly improved survival in diffuse large B-cell lymphoma (DLBCL) and became the global standard of care, establishing the proof-of-concept for antibody-based immunotherapy [49]. This milestone paved the way for subsequent generations of antibody-based therapies, including antibody-drug conjugates (e.g., brentuximab vedotin, polatuzumab vedotin) and bispecific antibodies, illustrating the iterative and cumulative nature of innovation in lymphoma therapeutics. The therapeutic landscape for aggressive B-cell lymphomas has been transformed by the introduction of CAR-T therapy and novel agents targeting specific molecular subtypes. The ability to move CAR-T therapy to earlier treatment lines has the potential to cure patients who would otherwise face poor outcomes with standard salvage approaches [50]. Figure 1 outlines the mechanisms of CAR T-cell therapy and bispecific antibodies, emphasizing their role in redirecting immune responses against malignant cells.

Antibody-drug conjugates have also shown significant promise in lymphoma treatment. Brentuximab vedotin has demonstrated activity in CD30-expressing lymphomas [51] while polatuzumab vedotin combined with rituximab and bendamustine improved overall survival in relapsed/refractory diffuse large B-cell lymphoma [52].

Precision medicine approaches in lymphoma increasingly rely on molecular subtyping to guide therapy selection. The identification of specific genetic alterations, such as MYC rearrangements, BCL2/BCL6 translocations, and mutations in genes such as TP53, helps inform prognosis and treatment decisions. Novel agents targeting specific pathways, including PI3K inhibitors and BCL-2 inhibitors, provide targeted options based on molecular characteristics [53]. Together, CAR T-cell therapy, antibody-drug conjugates, and molecular subtyping are redefining lymphoma care, moving toward personalized and potentially curative strategies. 

#### 2.6.4. Multiple Myeloma

Multiple myeloma has benefited from an extensive array of new therapeutic options, including immunomodulatory drugs, proteasome inhibitors, monoclonal antibodies, and, more recently, bispecific antibodies and CAR-T therapy. The sequential introduction of these agents has led to a substantial improvement in overall survival, with many patients now living for decades with their disease [54].

BCMA-directed therapies have shown particular promise, with both CAR-T cell therapy (idecabtagene vicleucel and ciltacabtagene autoleucel) and bispecific antibodies demonstrating high response rates in heavily pretreated patients. The challenge moving forward will be optimal sequencing of these therapies and development of strategies to overcome resistance mechanisms [55].

**Figure 2:**
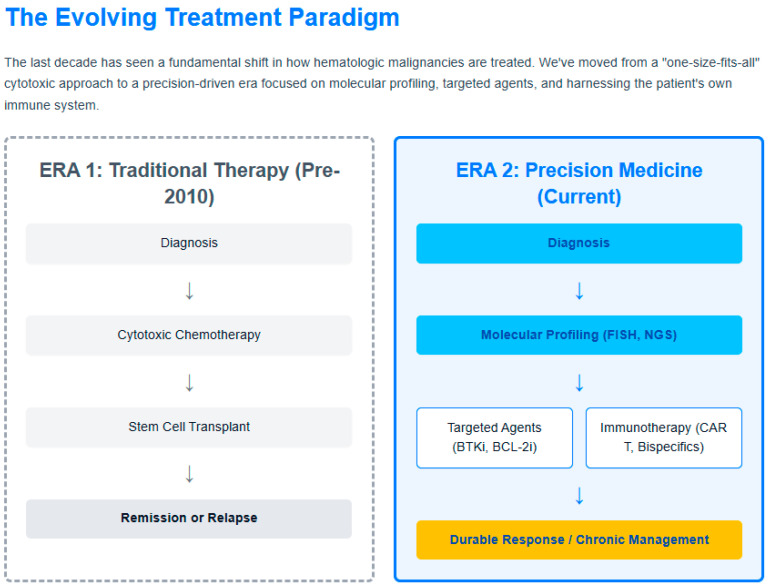
Figure 2 illustrates the shift from chemotherapy-based regimens to biomarker-driven, targeted, and immunotherapeutic approaches, reflecting the personalized treatment paradigm now central to hematologic oncology.

**Figure 3:**
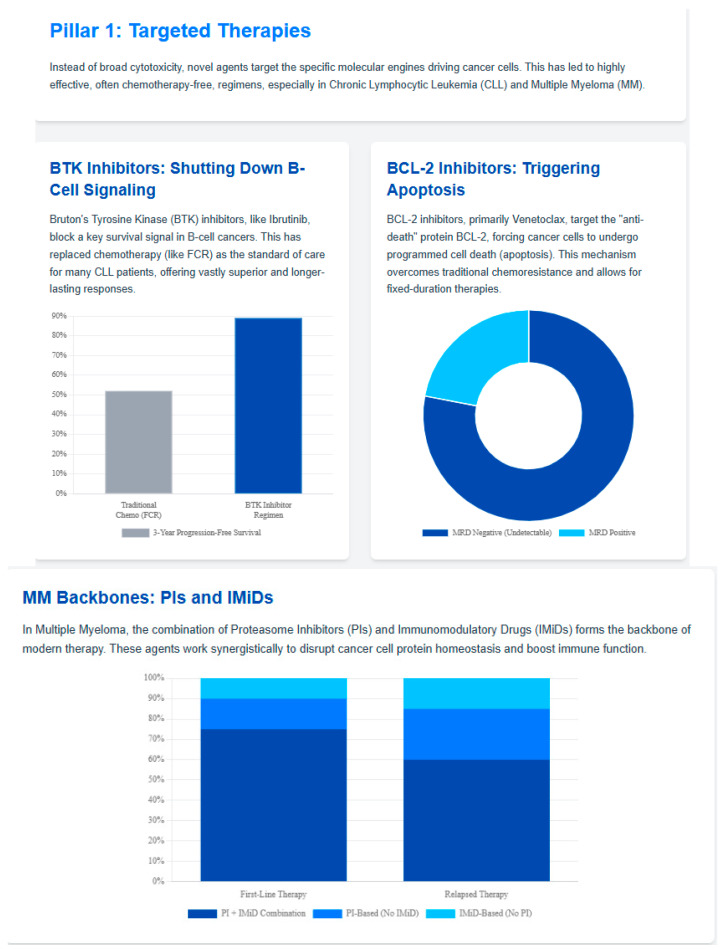
Figure 3 summarizes key targeted agents in CLL and multiple myeloma, highlighting the expansion of therapeutic options beyond traditional immunomodulatory drugs and proteasome inhibitors.

Novel targets beyond BCMA are being actively investigated, including GPRC5D and FcRH5 [56]. These alternative targets may provide options for patients with BCMA-negative disease or those who have developed resistance to BCMA-directed therapies. Early phase studies of GPRC5D-targeted bispecific antibodies have shown encouraging results, with response rates comparable to BCMA-targeted agents [57]. Figure 2 summarizes the evolving treatment paradigm, and Figure 3 is an infographic for targeted therapies. Table 1 summarizes the various novel therapies in these malignancies. The rapid succession of novel immunotherapies has markedly improved outcomes in myeloma, though optimal sequencing remains a key clinical challenge.

#### 2.6.5. Advances in Pediatric Hematologic Cancers

While this review focuses primarily on adult malignancies, pediatric hematologic cancers have also seen transformative advances, exemplified by the incorporation of blinatumomab and nelarabine in ALL regimens (e.g., ALLTOGETHER study) and the historical milestone of rituximab as the first therapeutic monoclonal antibody in lymphoma. Pediatric precision oncology initiatives such as INFORM, iTHER, and MAPPYact demonstrate the feasibility of molecular profiling to guide targeted therapy in children, though access barriers remain significant.

#### 2.6.6. Acute Lymphoblastic Leukemia (ALL)

The treatment landscape for B-cell acute lymphoblastic leukemia (B-ALL) has been transformed by immunotherapy, particularly in high-risk and relapsed/refractory disease. Blinatumomab, a bispecific T-cell engager targeting CD19, demonstrated superior outcomes compared to chemotherapy in relapsed/refractory B-ALL, leading to its approval in both adult and pediatric populations [58]. More recently, inotuzumab ozogamicin, an antibody-drug conjugate targeting CD22, has shown significant efficacy with higher rates of complete remission and minimal residual disease negativity compared to standard therapy in relapsed/refractory ALL [59]. These agents have been integrated into front-line and salvage regimens, including pediatric protocols such as the ALLTOGETHER study, which evaluates novel combinations in a risk-adapted manner [60]. The success of these immunotherapies underscores the shift from intensive, toxic chemotherapy to targeted immune-based strategies across all age groups.

### 2.7. Mechanistic Insights and Resistance Mechanisms

#### Understanding Therapeutic Resistance

The emergence of resistance to targeted therapies represents a critical challenge in hematologic malignancy treatment [61]. Resistance mechanisms are diverse and often involve mutations in target proteins, activation of alternative signaling pathways, or changes in the tumor microenvironment.

BTK inhibitor resistance primarily occurs through mutations at the C481 binding site, which disrupt the covalent binding of first-generation inhibitors [62]. However, resistance to non-covalent BTK inhibitors involves different mutations, including those at residues L528, M437, and T474 [63]. Venetoclax resistance involves multiple mechanisms, including upregulation of alternative anti-apoptotic proteins such as MCL-1, mutations in BCL-2 itself, and alterations in mitochondrial metabolism [41].

These insights have informed combination strategies aimed at preventing or overcoming resistance, such as the combination of venetoclax with MCL-1 inhibitors or metabolic modulators [40].

CAR-T cell resistance mechanisms include antigen loss or downregulation, T-cell exhaustion, and immunosuppressive microenvironmental factors [64]. Strategies to overcome CAR-T resistance include the use of multi-target CAR constructs and combination with immune checkpoint inhibitors or other immunomodulatory agents [65].

### 2.8. Biomarkers for Therapy Selection

The identification of predictive biomarkers has become increasingly important for optimal therapy selection in hematologic malignancies [66].

Genomic profiling at diagnosis can identify actionable mutations and inform treatment decisions, while monitoring of measurable residual disease provides insights into treatment response and relapse risk [67].

Liquid biopsies, including circulating tumor DNA (ctDNA) analysis, are emerging as valuable tools for treatment monitoring and resistance detection. In lymphoma, ctDNA clearance has demonstrated strong prognostic value and may serve as an earlier indicator of treatment efficacy compared to traditional imaging-based assessments [68].

Flow cytometry-based minimal residual disease monitoring has established prognostic significance in multiple disease contexts, like AML. Patients achieving undetectable MRD have superior outcomes across multiple treatment approaches, supporting the use of MRD as both a prognostic marker and potential surrogate endpoint in clinical trials [69].

### 2.9. Challenges and Controversies

#### 2.9.1. Treatment Sequencing and Combination Strategies

The abundance of active agents in hematologic malignancies has created new challenges related to optimal treatment sequencing and combination design. The question of when to use intensive versus targeted approaches, particularly in younger patients who may be candidates for either strategy, remains a subject of ongoing investigation [70].

In CLL, the choice between continuous BTK inhibitor therapy and fixed-duration venetoclax-based combinations involves considerations of treatment duration, long-term toxicity, and patient preference. Both approaches have demonstrated superior efficacy compared to chemoimmunotherapy, but direct comparative studies are needed to establish optimal sequencing [71].

#### 2.9.2. Toxicity Management and Quality of Life

While targeted therapies often have more favorable toxicity profiles compared to traditional chemotherapy, they are associated with unique side effects that require specialized management. CAR-T therapy-associated CRS and ICANS require intensive monitoring and may necessitate ICU-level care [8].

Standardized grading systems and treatment algorithms have improved outcomes, but toxicity remains a significant concern. Long-term effects of novel therapies are still being characterized, as many of these agents have only been in clinical use for a few years [8].

Secondary malignancies have been reported with some CAR-T products, highlighting the importance of long-term surveillance [8]. Similarly, the cardiovascular effects of BTK inhibitors require ongoing monitoring, particularly in older patients with multiple comorbidities [72].

Quality of life considerations are increasingly important in treatment decision-making, particularly as overall survival improves and patients live longer with their diseases [73].

Patient-reported outcome measures have shown that many targeted therapies maintain or improve quality of life compared to chemotherapy [74].

### 2.10. Evolution of Immunotherapeutic Modalities

The immunotherapy landscape has evolved from early monoclonal antibodies (e.g., rituximab) to antibody-drug conjugates (e.g., brentuximab vedotin, inotuzumab ozogamicin), bispecific T-cell engagers, and cellular therapies. Each modality leverages distinct mechanisms—from direct antigen targeting and payload delivery to immune cell redirection—contributing to the layered and increasingly personalized approach now standard in hematologic malignancies.

### 2.11. Clinical Implications and Future Directions

#### Personalized Medicine and Precision Oncology

The future of hematologic malignancy treatment will increasingly rely on comprehensive molecular profiling to guide therapy selection [75]. Whole-genome sequencing, RNA sequencing, and functional drug screening platforms are being developed to provide personalized treatment recommendations based on individual tumor characteristics [76].

Artificial intelligence and machine learning approaches are being applied to integrate multiple data types, including genomic, transcriptomic, and clinical data, to predict treatment responses and optimize therapy selection. These approaches may help predict resistance mechanisms before they become clinically apparent [77].

The development of patient-derived models, including organoids and xenografts, provides opportunities for functional testing of treatment approaches and combination strategies. These models may be particularly valuable for rare disease subtypes where clinical trial enrollment is challenging [78].

## 3. Novel Targets and Therapeutic Approaches

Emerging therapeutic targets in hematologic malignancies include epigenetic modifiers, metabolic enzymes, and components of DNA damage response pathways [79].

MALT1 protease inhibitors have shown promise in preclinical models of B-cell malignancies and may provide an alternative to BTK inhibition or serve as combination partners [80].

Immune-based approaches beyond CAR-T therapy are being actively investigated, including bispecific T-cell engagers, natural killer cell therapies [81,82]. These approaches may provide alternatives for patients who are not candidates for CAR-T therapy or as combination partners to enhance efficacy [82].

Gene editing approaches, including CRISPR-Cas9, are being explored both as therapeutic interventions and as tools to enhance existing therapies. In situ gene editing of T-cells could potentially improve CAR-T therapy by enhancing persistence, reducing exhaustion, or incorporating multiple target specificities [83,84,85].

### Global Access and Health Economics

The high cost of many novel therapies in hematologic malignancies raises important questions about global access and health economics. CAR-T therapies can cost several hundred thousand dollars per treatment course [86]. Strategies to improve access include the development of simplified manufacturing processes for cellular therapies [87], biosimilar versions of expensive biologics [88], and risk-sharing agreements between manufacturers and health systems [89].

**Figure 4:**
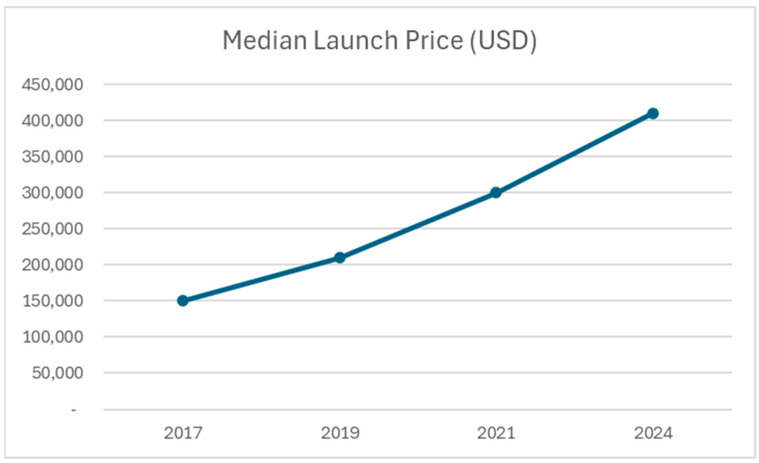
Figure 4 highlights the significant cost burden of novel therapies, underscoring the need for value-based pricing and improved access strategies.

International cooperation and technology transfer may help extend access to novel therapies in resource-limited settings [90]. Value-based pricing models that tie reimbursement to clinical outcomes may help align costs with patient benefit while incentivizing continued innovation. Outcome-based contracts and real-world evidence generation will likely play increasingly important roles in drug pricing and reimbursement decisions [89]. Figure 4 shows the median launch price in US dollars for these agents.

## 4. Future Research Directions

### 4.1. Combination Therapy Development

Future clinical trials will need to address fundamental questions about optimal combination strategies, including the selection of rational partners, appropriate dosing and scheduling, and patient selection criteria [34]. Adaptive trial designs may be particularly valuable for evaluating multiple combinations efficiently while minimizing patient exposure to ineffective regimens [91]. In vivo CAR-T manufacturing using lipid nanoparticles remains a promising but challenging frontier, requiring resolution of delivery efficiency and immunogenicity concerns.

Biomarker-driven combination selection represents a promising approach for improving therapeutic indices. The identification of predictive markers for combination benefit could help select patients most likely to benefit from specific multi-agent regimens while avoiding unnecessary toxicity in others [92]. Optimal sequencing of CAR T-cell therapy and bispecific antibodies—particularly in relapsed myeloma and lymphoma—warrants prospective comparative studies.

Early phase combination studies should incorporate comprehensive correlative analyses to understand mechanisms of synergy and resistance. These studies will be critical for identifying biomarkers of response and developing strategies to overcome resistance [93].

### 4.2. Emerging Technologies

Advances in manufacturing and delivery technologies may help reduce costs and improve access to cellular therapies [94]. In vivo CAR-T generation using lipid nanoparticles could potentially eliminate the need for complex ex vivo manufacturing processes [94]. Digital health technologies, including wearable devices and smartphone applications, may enhance the monitoring of patients receiving novel therapies and enable earlier detection of toxicities. These technologies could be particularly valuable for managing patients receiving CAR-T therapy or other treatments associated with multiple side effects [95]. Advances in imaging and liquid biopsy technologies will likely provide more sensitive methods for treatment monitoring and response assessment [96]. Emerging MRD technologies, including high-sensitivity flow cytometry, next-generation sequencing, and ctDNA assays, are poised to refine response assessment and guide treatment duration.

## 5. Regulatory Considerations

The regulatory landscape for hematologic malignancy therapeutics will need to evolve to accommodate increasingly complex therapeutic approaches, including combination regimens and personalized medicine strategies [97]. Adaptive trial designs and master protocol approaches may help streamline the development and approval of novel combination therapies [98,99].

Real-world evidence generation will play an increasingly important role in supporting regulatory decisions and informing clinical practice [100]. Post-marketing studies will be essential for understanding long-term safety and efficacy of novel therapies in diverse patient populations [101].

International harmonization of regulatory standards and approval processes could help ensure more equitable global access to novel therapies while maintaining appropriate safety and efficacy standards [102].

## 6. Conclusions

The therapeutic revolution in hematologic malignancies marks a paradigm shift that has fundamentally redefined clinical practice and patient expectations. The decisive shift from generalized cytotoxic regimens to highly specific, targeted therapies and potent immunotherapies has unlocked unprecedented rates of deep, durable remission across a spectrum of blood cancers. While these advances offer immense promise, the path forward requires sustained focus on several critical areas. Specifically, future efforts must concentrate on optimizing next-generation combination regimens, comprehensively understanding and circumventing mechanisms of acquired drug resistance, and ensuring equitable access to these high-cost, life-extending treatments by addressing financial burden. Continued translational research into novel molecular targets and the adoption of dynamic, adaptive clinical trial designs will be crucial to further personalize therapy, refine treatment duration, and ultimately transition more hematologic cancers towards increased remission rates and disease-free survival.

## Figures and Tables

**Table 1:** Major Novel Agents and Therapeutic Impact in Key Hematologic Malignancies.

Target Malignancy	Drug Class	Example Agents (First/Second Gen)	Mechanism of Action (Molecular Target)	Key Clinical Impact
Chronic Lymphocytic Leukemia (CLL)	BTK Inhibitors	Ibrutinib, Acalabrutinib, Zanubrutinib	Covalent/Non-Covalent inhibition of Bruton’s Tyrosine Kinase (BTK)	Chemotherapy-free regimens, high response rates in high-risk groups (e.g., del(17p))
	BCL-2 Inhibitors	Venetoclax	Selectively inhibits the anti-apoptotic protein BCL-2	Enables fixed-duration therapy, overcomes chemoresistance
Multiple Myeloma (MM)	IMiDs (Immunomodulatory Drugs)	Lenalidomide, Pomalidomide	Binds to Cereblon (CRBN), leading to degradation of transcription factors	Backbone of treatment (induction and maintenance), synergistic with proteasome inhibitors
	Proteasome Inhibitors (PIs)	Bortezomib, Carfilzomib, Ixazomib	Inhibits the 20S core particle of the proteasome	Induces apoptosis via accumulation of toxic misfolded proteins
B-cell Non-Hodgkin Lymphoma (B-NHL)	CAR T-cell Therapy	Axicabtagene Ciloleucel, Tisagenlecleucel	Redirects patient’s T-cells to express chimeric antigen receptor (targeting CD19)	Curative potential in relapsed/refractory disease
	Bispecific Antibodies	Mosunetuzumab, Epcoritamab	Engages CD3 on T-cells and CD20/CD38 on cancer cells (T-cell redirection)	“Off-the-shelf” alternative to CAR T-cells, targeting specific antigens
Acute Myeloid Leukemia (AML)	IDH Inhibitors	Ivosidenib (IDH1), Enasidenib (IDH2)	Blocks mutant Isocitrate Dehydrogenase (IDH) enzyme, promoting differentiation	Targeted treatment for specific molecular mutations

## Data Availability

No new data were created or analyzed in this study.
